# Grocery food taxes and U.S. county obesity and diabetes rates

**DOI:** 10.1186/s13561-021-00306-2

**Published:** 2021-02-13

**Authors:** Lingxiao Wang, Yuqing Zheng, Steven Buck, Diansheng Dong, Harry M. Kaiser

**Affiliations:** 1 Department of Agricultural Economics, University of Kentucky, Lexington, KY 40546 USA; 2 Department of Agriculture, Economic Research Service, U.S, Washington, DC 20024 USA; 3 The Charles H. Dyson School of Applied Economics and Management, Cornell University, Ithaca, NY 14853 USA

**Keywords:** Grocery tax, Diabetes, Obesity

## Abstract

**Background:**

Grocery food taxes represent a stable tax revenue stream for state and municipal government during times of adverse economic shocks such as that observed under the coronavirus disease 2019 (COVID-19) pandemic. Previous research, however, suggests a possible mechanism through which grocery taxes may adversely affect health. Our objectives are to document the spatial and temporal variation in grocery taxes and to empirically examine the statistical relationship between county-level grocery taxes and obesity and diabetes.

**Methods:**

We collect and assemble a novel national dataset of annual county and state-level grocery taxes from 2009 through 2016. We link this data to three-year, county-level estimates based on data from the Centers for Disease Control and Prevention on rates of obesity and diabetes and provide a nation-wide spatial characterization of grocery taxes and these two health outcomes. Using a county-level fixed effects estimator, we estimate the effect of grocery taxes on obesity and diabetes rates, also controlling for a subset of potential confounders that vary over time.

**Results:**

We find a 1 percentage point increase in grocery taxes is associated with 0.588 and 0.215 percentage point increases in the county-level obesity and diabetes rates.

**Conclusion:**

Counties with grocery taxes have increased prevalence of obesity and diabetes. We estimate the economic burden of increased obesity and diabetes rates resulting from grocery taxes to be $5.9 billion. Based on this estimate, the benefit-cost ratio of removing grocery taxes across the United States only considering the effects on obesity and diabetes rates is 1.90.

**Supplementary Information:**

The online version contains supplementary material available at 10.1186/s13561-021-00306-2.

## Background

Grocery sales taxes (hereafter referred as grocery taxes) are sales taxes imposed on grocery foods and exist in the form of a state tax, a county tax, or both in sixteen U.S. states. Taxing groceries is an attractive revenue source for state and municipal governments because grocery sales are relatively stable; thus, facilitating budgeting planning even during times of economic downturn. Of course, grocery taxes make grocery foods more expensive, which society may feel most during times of economic downturn as lower income households become even more food insecure. For example, coronavirus disease 2019 (COVID-19) pandemic began in early 2020, food insecurity sky-rocketed in the United States—in April 2020, food insecurity increased to 23%.^1^ Not surprisingly, food insecurity is associated with social problems (particularly for children) such as health [1–3], psychological [4], and behavioral problems [5, 6]; therefore, policies thought to impact food insecurity and health have been extensively studied. Notably, there are studies that have analyzed the impacts of specific food taxes, such as soda taxes, on consumption and health. Recent examples include studies showing that at-risk subpopulations such as obese children coming from low-income families are more sensitive to soda taxes [7, 8].

In contrast, the relationship between grocery taxes and health outcomes has received little attention. This is somewhat surprising given that relative to soda taxes, grocery taxes are far more common, a significantly larger percentage tax on average, and they apply to all grocery foods so represent a considerably larger share of household income. The current lack of research on the impacts of grocery taxes is unfortunate since it is during times of economic hardship, such as a COVID-19 induced recession, that policies such as grocery taxes receive greater consideration as a source of stable tax revenue for state and local governments.

Grocery taxes can affect the odds of eating at home versus dining out through changing the relative effective prices (tax included price) of grocery and restaurant foods. Compared with states such as New York where restaurant foods are taxed while grocery foods are tax exempt, taxing both grocery and restaurant foods in states like Alabama creates more of a disincentive to eat at home [9]. For the poorest segment of the population, fast food restaurants become their primary option as a substitute for grocery foods because fast food restaurants are both more accessible [10, 11] and cheaper [12]. In particular, two recent empirical studies show that grocery taxes reduced U.S. consumers’ grocery food expenditures and increased restaurant food expenditure, and restaurant food sales taxes increased U.S. consumers’ grocery food expenditures [13, 14]. Therefore, the substitution from grocery food to fast food in response to taxing groceries may increase the odds of unhealthy outcomes since there is evidence that consumption of fast food affects a person’s risk of becoming both obese [15] and diabetic [16].

Unlike soda or fat taxes, grocery taxes apply to thousands of grocery items and may effectively change consumers’ grocery food choices. Though not all grocery foods are healthy, reduced consumption of fruits and vegetables may induce obesity [17] and diabetes [18], and food-at-home is widely considered healthier than food-away-from-home. Therefore, we hypothesize that health outcomes are negatively correlated with grocery taxes. We choose two health outcome measures for this study: obesity and diabetes rates within a county, because food consumption is closely related to obesity and diabetes.

It is well known that individuals gain weight whenever consumed calories exceeds expended calories [19]. Yet, rates of obesity vary significantly from person to person according to the individual’s social economic status [20] like education [21], income [22], gender [23], age, and race [24]. In addition, individual body mass index (BMI) is also highly related with individual risky behavior such as smoking [25] and alcohol consumption [26]. However, these individual-level reasons do not explain fully the increasing prevalence of obesity across the entire society over time.

Researchers from multiple disciplines have identified various underlying causes of obesity epidemic from different perspectives, such as decreasing price per calorie [17], high availability of fast food, high cost of healthy food [27], difficulty to access healthy food especially for lower-income households [28], and the high amount of marketing of unhealthy food and beverages especially among younger children [29]. While the evidence is mixed, some studies have identified physical inactivity as a cause for obesity, attributed to urban sprawl [30], labor-saving devices such as dish washers [31], and increasingly sedentary occupations [32]. Similar to findings in the obesity literature, the rising rates of diabetes has been attributed in part to environmental factors, such as the abundance of food supply and sedentary lifestyles [33–35]. In fact, 60% of diabetes cases can be attributed to being obese or overweight [36].

In terms of magnitude, the quantitative significance for obesity and diabetes risk factors also varies widely. For instance, quitting smoking has been found to reduce body mass index (BMI) by 1.8–1.9 units with a BMI above 30 defining obesity [25]. As a separate example, a one percent increase in soda taxes has been associated with at 0.013 decrease in average BMI [8]. Overall, there is not clear consensus on the aggregate effects of different risk factors on either obesity or diabetes rates, especially among individual studies that examine specific sub-populations.

In summary, the public health literature has identified a multitude of causes for the rising obesity and diabetes epidemic in the United States, including prices, food availability and accessibility, and marketing. The aim of this study is to examine another potential factor which has not been investigated previously: the relationship between grocery food taxes and health outcomes. Despite the fact that groceries are taxed in one third of U.S. states as well as on-going debates on whether to impose significant grocery taxes (e.g., New Mexico and West Virginia), there is, to our knowledge, no comprehensive dataset on state and county-level grocery taxes exists. Therefore, one contribution of our work is the development of a comprehensive dataset on state and county-level grocery taxes from 2009 through 2016, which we then link to county-level estimates of obesity and diabetes rates. The main empirical contribution of our work is to estimate the effect of grocery taxes on these two important health outcomes using our novel county-level panel data and a county fixed effects estimator that also includes time-varying variables to control for socioeconomic factors, risky behaviors, and food access and affordability environment. A third contribution is policy-focused, we calculate benefit-cost ratios of eliminating grocery taxes as a way to assess the quantitative significance of grocery taxes in determining obesity and diabetes rates.

## Methods

### Data organization and structure

**Fig. 1:**
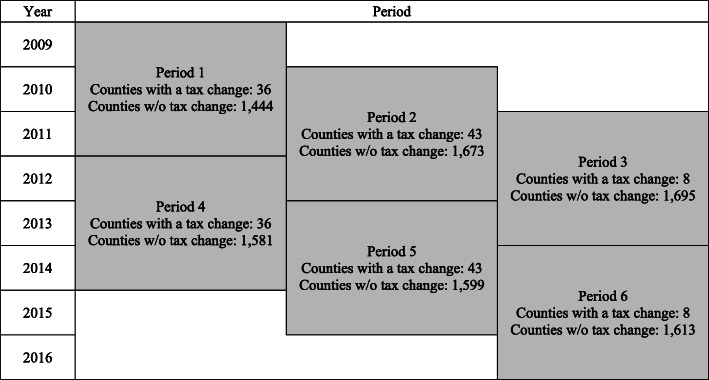
Study Design

We organize county-level panel data consisting of six time periods on obesity and diabetes rates, food taxes, socioeconomic characteristics, and risky health behaviors. Each of the six periods is 3 years in length; thus, the unit of observation in the statistical analysis is the county-three-year period. Each of the six periods in the study has a 1 year overlap with the subsequent period or the preceding period or both—Fig. 1 depicts this somewhat unique structure of our county-level panel data and empirical design. We develop this data structure because the outcome variables of obesity and diabetes rates are only precisely estimated and reported based on the average of a 3 year-sample window. Concordance on the timing of measurements between the health outcome variables and the explanatory variables requires that the food tax, socioeconomic, and risky health behavior variables also be measured as three-year averages. A separate justification for measuring each variable as a three-year average is that the adjustment of diets due to a tax change, and any subsequent transition to or from obesity is not likely immediate.

### Data sources

We assemble a large set of data on state- and county-level grocery tax rates in the U.S. from 2009 to 2016. The key independent variable of interest in this study is the total grocery sales tax, measured as a percentage. The total tax is the sum of the state-level and county-level grocery sales taxes. We also collect data on restaurant sales taxes, which we use to calculate the ratio of the grocery to restaurant sales tax as an alternative explanatory variable. The tax data are obtained from Bridging the Gap for state tax rates, Tax-Rates.org for 2016 county rates, and state Departments of Revenue for the rest (by online searching by two research assistants over an extended period of time).

We assess two dependent variables in our analyses: 1) three-year county-level obesity prevalence; and 2) three-year county-level diabetes prevalence. County-level rates of diagnosed obesity and diabetes are obtained from the Centers for Disease Control and Prevention (CDC) county data indicators [37], which are three-year average rates calculated by CDC using annual surveys from the Behavioral Risk Factor Surveillance System (BRFSS) [38] and are based on a three-year average to improve precision. For both obesity and diabetes outcomes we use age-adjusted rates to measure the health outcomes.

We collect data for control variables in the regression analysis from multiple sources on a wide range of socioeconomic data measured at the annual level. To conform the explanatory variables with the dependent variable, we use the annual socioeconomic data to construct three-year county level averages for use as control variables in the regression analysis. The first set includes food environment/access/affordability including the numbers of grocery stores, fast food restaurants, and full-service restaurants, and the average cost per meal. The former three variables are from the Census Bureau’s County Business Patterns [39] and the latter is from Feeding America [40]. Socioeconomic measures on population, race, gender income, employment and education are based on data from the Census Bureau’s Population Estimates Program [41]. The per capita income and employment rate are from the Regional Economic Information System (REIS) [42].

Additional control variables include data on risky health behaviors, which are also at the annual level and used for constructing three-year county-level averages. The county-level prevalence estimates of smoking and alcohol use are obtained from BRFSS. Smoking is measured as the percentage of adults in a county who both report that they currently smoke every day or most days and have smoked at least 100 cigarettes in their lifetime. Excessive alcohol use is the percentage of adults that report excessive alcohol consumption in the past 30 days in each county. Data on drug-possession and driving under the influence (DUI) arrests are obtained from the County Level Detailed Arrest and Offense Data supported by the Uniform Crime Reporting (UCR) Program [43]. We divide the arrests by county population from REIS to obtain per capita possessing-drug and DUI arrests.

In total we have tax data for 3101 U.S. counties. We only keep 2446 counties in the dataset due to our study design. Moreover, 408 counties are lost when merging in socioeconomic variables. After eliminating the 180 singleton counties, we are left with 1858 counties in the dataset. Of these counties, 87 experienced a grocery tax change in the year 2012, 2013 or 2014. The other 1771 counties experienced no grocery tax change during the study window (2009–2016); 1250 of these counties never have a grocery tax, while 521 have a constant grocery tax during the study window. In terms of our entire panel of county-period observations, we only keep observations for which the grocery tax is constant within the three-year period. As a consequence, counties with grocery tax changes appear in exactly two periods each, which correspond to either 1) the 3 year periods before and after 2012, 2) the 3 year periods before and after 2013, or 3) the 3 year periods before and after 2014. Counties with no tax grocery tax changes during our study window will appear in each of the six periods unless there is missing data for covariates in a county for some years. If our panel of counties with no tax changes is balanced, then we would have 11,148 observations (1858 counties by 6). Of the 1771 counties without tax changes, 1319 of them appear in all six periods. In terms of total county-period observations, we have 9979 observations; observations 9805 observations from our panel of counties that never experience a tax change and 174 observations from counties that do experience a tax change.

### Statistical analysis

We estimate the effects of grocery taxes on obesity and diabetes rates resulting from changes in county-level grocery taxes in the years 2012, 2013 and 2014. Our estimating procedure uses a county fixed effects linear regression model for county-level, age-adjusted health outcomes. The main explanatory variables of interest are 1) grocery taxes and 2) restaurant taxes. Our main parameter of interest describes how changes in the county-level total grocery sales tax relates to county-level health outcomes on average, after parsing out other observable variables and unobservable time-constant variables. Standard errors are clustered at the state level to account for arbitrary intra-cluster correlations between the error terms [44]. The regression model controls for county-level food access, demographics, socioeconomics, and risky health behaviors. The model also includes period fixed effects to control for period-specific time shocks common to all counties and county fixed effects to control for county-specific time-invariant factors.

In addition to the main analysis described above, we assess the robustness of our results to an alternative measure of food taxes—the ratio of the grocery tax to the restaurant tax. Because some counties have no restaurant tax, we add 0.01 to both the numerator and denominator. This adjustment has only a small influence on the ratio when then the restaurant tax is non-zero, which is the vast majority of observations. In all instances when the restaurant tax is zero, the grocery tax is also zero, which makes the ratio equal to one in such cases. To us this transformation is reasonable since it keeps intact the ratio when the denominator is non-zero, and implies parity when the denominator is zero.

## Results

### A map of grocery taxes

**Fig. 2:**
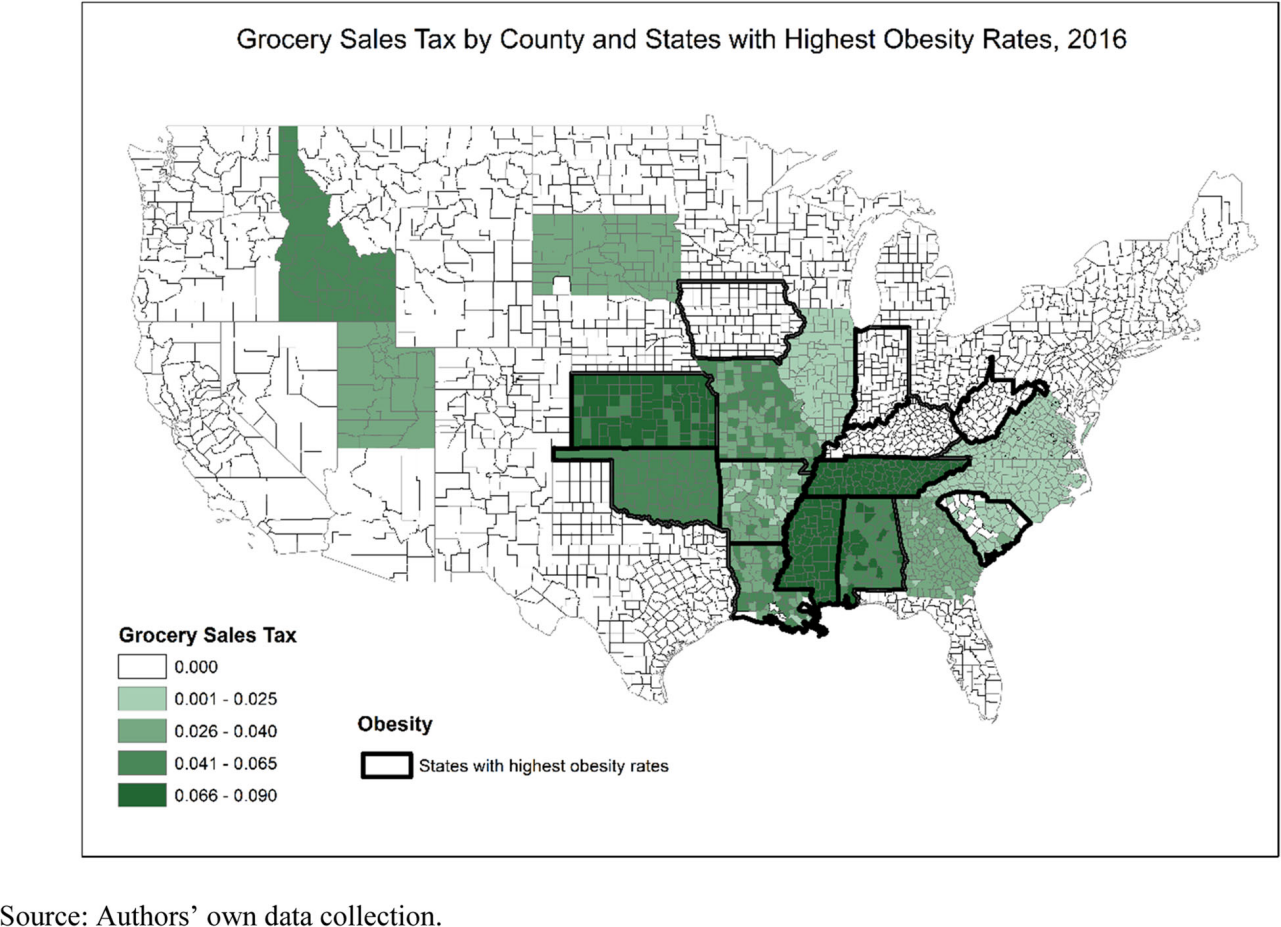
U.S. Grocery Sales Tax Distribution for the Year of 2016

Figure 2 presents a map of the United States depicting county-level grocery taxes along with the top 12 most obese states identified in bold. This figure illustrates that grocery taxes are more prevalent in states with the highest obesity rates.

### Health outcomes by taxing status

**Fig. 3:**
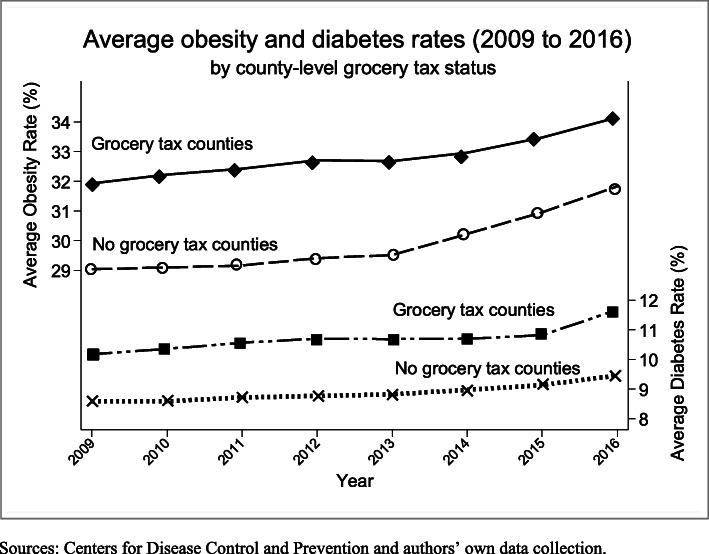
Average Obesity and Diabetes Rates by Grocery Tax

Figure 3 plots the average rates of obesity and diabetes from 2009 through 2016 for both counties with and without a grocery tax (state, county, or both). Over this period, the national average obesity and diabetes rates increased significantly, especially after 2013. If we look at counties with and without grocery sales tax separately, the taxed counties are less healthy. Specifically, the average obesity and diabetes rates of counties with taxes are approximately 3 and 2.5 percentage points higher, respectively. Figure 3 clearly shows that counties with a grocery tax consistently were worse for both obesity and diabetes.

### Regression results on obesity and diabetes rates

In Table 1 we present the summary statistics of the variables used in our analysis. The first three columns in Table 2 report the regression results of obesity rates on grocery sales tax rates under a base specification with year fixed effects, the base specification augmented with county fixed effects, and a third specification that also adds time-varying control variables. The results in columns 1, 2 and 3 are all similar with point estimates of 0.707, 0.606 and 0.588, respectively. Under all specifications, the grocery tax is positive and statistically significant at the 1% level. Our preferred specification reported in column 3, which includes the most comprehensive controls (county fixed effects plus a number of factors identified in the literature), suggests that a one-percentage point increase in the grocery tax rate is associated with a 0.588 percentage point increase in the obesity rate. In contrast, the coefficient on the restaurant tax is negative in sign (− 0.158), though it is statistically indistinguishable from zero.

**Table 1:** Summary Statistics of the Variables Used

	Unit	Mean	S.D.	Min	Max
*Health Outcomes*					
Obesity rate (age-adjusted diagnosed)	%	30.419	4.84	10.7	47.6
Diabetes rate (age-adjusted diagnosed)	%	9.305	2.122	3.4	19.4
*Tax Variables*					
Total grocery sales tax rate	%	1.142	2.084	0.000	9.000
Total restaurant sales tax rate	%	6.036	1.686	0.000	9.933
(1 + Grocery Tax)/(1 + Restaurant Tax)		0.340	0.328	0.093	1.000
*Socioeconomic Variables*					
Grocery stores per capital	1/1000	0.221	0.14	0.017	1.701
Fast-food restaurants per capital	1/1000	0.616	0.198	0.044	1.964
Full-service restaurants per capital	1/1000	0.781	0.414	0.042	3.995
Cost per meal	$	2.775	0.306	1.956	5.113
White		0.857	0.145	0.093	0.991
Black		0.087	0.133	0.000	0.85
Female		0.502	0.016	0.366	0.553
Hispanic		0.085	0.12	0.004	0.957
Income per capita	1000$	39.014	10.899	18.768	199.241
Employees’ share of total population		0.527	0.144	0.219	3.213
Share of bachelor’s degree or higher of the 25-year- and-over population	%	21.983	9.291	5.967	72.867
Smoking rate	%	20.599	5.096	3.167	42.160
Drinking rate	%	15.331	4.947	1.6	35.933
Drug arrest rate		0.005	0.04	0.000	1.893
DUI		0.006	0.034	0.000	1.886
Counties: 1858; Obs.: 9779

**Table 2:** Regression Results of Health Outcomes on Respective Grocery and Restaurant Sales Taxes

Dependent variable: Obesity Prevalence (unit: %, mean: 30.419, S.D.: 4.840) Diabetes Prevalence (unit: %, mean: 9.305, S.D.: 2.122)						
	Obesity			Diabetes		
	1	2	3	4	5	6
Total Grocery Sales Tax Rate (%)	0.707***	0.636***	0.588***	0.400***	0.252**	0.215**
	(0.203)	(0.153)	(0.154)	(0.118)	(0.108)	(0.098)
Total Restaurant Sales Tax Rate (%)	0.369*	−0.147	−0.158	0.290***	−0.134	− 0.127
	(0.194)	(0.139)	(0.127)	(0.096)	(0.111)	(0.101)
Observations	9779	9779	9779	9779	9779	9779
R-squared	0.129	0.909	0.910	0.227	0.927	0.928
Period FE (m_period = 6)	Y	Y	Y	Y	Y	Y
County FE (m_county = 1858)			Y			Y
Controls		Y	Y		Y	Y

Note: Standard errors are in parentheses. *, **, and *** denote statistically significance at the 10, 5, and 1% levels, respectively

The results in columns 4, 5 and 6 present the results for diabetes rates. The point estimates of 0.400, 0.252 and 0.215, respectively. Under all specifications, the grocery tax is positive and statistically significant at the 5% level. Our preferred specification reported in column 6 suggests that a one-percentage point increase in the grocery tax rate is associated with a 0.215 percentage point increase in the diabetes rate. Again, the coefficient on the restaurant tax is negative in sign (− 0.127), though it is statistically indistinguishable from zero.

In Table 3 we assess the robustness of our results for both obesity and diabetes rates using an alternative food tax measure—the grocery tax to restaurant tax ratio is used as the main independent variable instead of the grocery tax. Results are consistent with those reported in Table 2 and are statistically significant at the 5% level for specifications including county fixed effects.

**Table 3:** Regression Results of Health Outcomes on Grocery to Restaurant Sales Taxes Ratio

Dependent variable: Obesity Prevalence (unit: %, mean: 30.419, S.D.: 4.840) Diabetes Prevalence (unit: %, mean: 9.305, S.D.: 2.122)						
	Obesity			Diabetes		
	1	2	3	4	5	6
Tax Ratio: (1 + Grocery Tax)/(1 + Restaurant Tax)	2.639	5.144***	4.760***	1.498	1.603***	1.296**
	(1.721)	(1.263)	(1.169)	(1.026)	(0.594)	(0.571)
Observations	9779	9779	9779	9779	9779	9779
R-squared	0.036	0.909	0.910	0.028	0.927	0.928
Period FE (m_period = 6)	Y	Y	Y	Y	Y	Y
County FE (m_county = 1858)			Y			Y
Controls		Y	Y		Y	Y

Note: Standard errors are in parentheses. *, **, and *** denote statistically significance at the 10, 5, and 1% levels, respectively

## Discussion

We find evidence that grocery taxes have an adverse effect on both obesity and diabetes rates. Specifically, assuming our county fixed effects estimator is not biased by time-varying omitted variables, then a one percentage point increase in grocery taxes increases obesity and diabetes rates by 0.588 and 0.215 percentage points, respectively.

To put our results in context from a policy perspective, we calculate benefit-cost ratios (BCRs) to summarize whether the health benefits associated with reducing the grocery tax by one percentage point are likely to exceed the cost of foregone tax revenues from their reduction. Table 4 reports the ratios and Additional File 1 shows the full regression results as well as the detailed steps to obtain the ratios.

**Table 4:** Summary of Aggregate U.S. Health Burdens and Benefit-Cost Ratios with Sensitivity Analysis

	1	2	3
	Obesity	Diabetes	Total
Aggregate U.S. health burdens (billions of USD)			
Low estimate	1.34	2.64	3.98
	(0.65, 2.03)	(0.56, 5.00)	(1.21, 7.03)
Preferred estimate	2.06	3.8	5.86
	(1.00, 3.11)	(0.81, 7.19)	(1.81, 10.30)
High Estimate	2.79	5.24	8.03
	(1.36, 4.23)	(1.11, 9.92)	(2.47, 14.15)
Benefit-cost ratios (health benefits / cost of reduced tax revenue)			
Low estimate	0.434	0.855	1.289
	(0.211, 0.657)	(0.091, 1.619)	(0.302, 2.276)
Preferred estimate	0.666	1.23	1.896
	(0.324, 1.008)	(0.163, 2.329)	(0.487, 3.337)
High Estimate	0.905	1.696	2.601
	(0.440, 1.369)	(0.181, 3.212)	(0.621, 4.581)

Note: In parentheses we report the 95% confidence interval derived from the sampling variability of the regression coefficients reported in columns (3) and (6) of Table 2

Our preferred estimates of annual expenditures (direct costs only) for treating obesity and diabetes are $1901 [45]. We also considered variations among cost estimates; for example, a meta-analysis found that the annual medical expenditures attributable to treating obesity for a person with the condition varies from $1239 to $2582 [46]. Therefore, in a sensitivity analysis we consider low and high estimates for these figures, these results are also summarized in Table 4.

The top portion of Table 4 summarizes our estimates of health burdens associated with grocery taxes. The aggregate U.S. health burden of grocery taxes in the year 2016 due to medical expenditures on obesity and diabetes is calculated to be $5.86 billion (95% C.I. is $1.81 billion to $10.30 billion).

The bottom portion of Table 4 summarizes the BCRs. The calculated BCRs for obesity, and diabetes using our preferred estimates of medical expenditures are 0.666 (95% C.I. is 0.324 to 1.008) and 1.23 (95% C.I. is 0.163 to 2.329), respectively. The BCR of these two factors combined is 1.896. Similar to health burden analysis, we also summarize the results of our sensitivity analysis for the BCR. Based on the sensitivity analysis and taking into account a range based on sampling variability of our regression output, our lowest estimate of the combined BCR is 1.289, and the highest is 2.601.

Many states and local municipalities have recently considered changing their grocery tax, such as West Virginia in 2017 (proposing an 8% new tax) and Utah in 2018 (proposing removing grocery taxes). States and counties that tax food need to understand that this policy is associated with adverse health outcomes. Our preliminary results suggest that officials in states that tax groceries should take a closer look at ways to lessen the potential burden of such taxes as a way to improve health outcomes for the community. Decreasing the grocery tax, would reduce tax revenue, and government officials would need to look at alternative revenue generating options if it lowered grocery taxes. Another option to off-set the potential adverse effects of grocery taxes would be a tax credit, though it would have to be sufficiently large to off-set the tax. Further, it is not clear how a lump-sum tax credit would affect the marginal responses to taxes we estimate in our analysis.

Furthermore, we find that the ratio of the grocery tax to the restaurant sales tax is also positively associated with adverse health outcomes. In particular, a doubling of this tax ratio is found to increase obesity and diabetes rates by an average of 0.773 and 0.21 percentage point, respectively. This has policy implications that should be considered especially by states and counties that are either considering levying a grocery tax or eliminating it. It is possible the adverse health outcomes could be lessened if this relative tax ratio were lowered in states with grocery taxes. For example, one option would be to consider a revenue neutral simultaneous decrease in the grocery tax and increase in the restaurant (particularly fast-food establishments) tax as a way to lessen adverse health outcomes.

## Conclusion

Our county-level depiction of grocery taxes in the United States reflects the first comprehensive dataset on state and county-level grocery taxes and shows a clear spatial correlation between grocery taxes and nutrition-related health outcomes. The regression results, which are based on data county fixed effects estimator, shows a strong statistical relationship between grocery taxes and both obesity and diabetes. Several states and counties are actively considering the levying or removal of grocery taxes. Our study design is only one component of the costs (or benefits) of a grocery tax; nonetheless, the results are thought-provoking and suggest the possibility of a large health burden from grocery taxes and a benefit-cost ratio greater than one corresponding to reductions in the grocery tax. Based on our findings using a novel panel dataset combining comprehensive county-level grocery tax data with county-level health outcome measures, we recommend both researchers and policy makers give further consideration to the removal of grocery taxes a possible mechanism to improve health outcomes. Meanwhile, more evidence would be required to pin down a mechanism through which grocery taxes may affect health outcomes; for example, more evidence on the potential link through fruit and vegetable consumption choices.

## Supplementary Information

**Additional file 1. A1.** Full Regression Results Health Outcomes on Grocery and Restaurant Sales Taxes. **A2.** Calculation of Health Burden and Benefit-Cost Ratio.

## Data Availability

The obesity and diabetes datasets generated during and/or analyzed during the current study are available in the CDC WONDER system. The scanner data that support the findings of this study are available from the Nielsen company but restrictions apply to the availability of these data, which were used under license for the current study, and so are not publicly available. The tax datasets used during the current study are available from the corresponding author on reasonable request.

## Abbreviations

COVID-19: Coronavirus disease 2019
BMI: Body mass index
CDC: Centers for Disease Control and Prevention
BRFSS: Behavioral Risk Factor Surveillance System
REIS: Regional Economic Information System
UCR: Uniform Crime Reporting
DUI: Driving under the influence
BCR: Benefit-cost ratio

## Footnotes

1 Source: https://www.brookings.edu/blog/up-front/2020/05/06/the-covid-19-crisis-has-already-left-too-many-children-hungry-in-america/
