# Supplementary material for: Grocery food taxes and U.S. county obesity and diabetes rates
Source: Health Econ Rev. 2021 Feb 13;11:5. doi: 10.1186/s13561-021-00306-2 (PMC7882053; doi:10.1186/s13561-021-00306-2)
Supplement: Supplementary file 1 — Additional file 1. A1. Full Regression Results Health Outcomes on Grocery and Restaurant Sales Taxes. A2. Calculation of Health Burden and Benefit-Cost Ratio. [file 13561_2021_306_MOESM1_ESM.docx]

**Additional File 1**

**A1.** Full Regression Results Health Outcomes on Grocery and Restaurant Sales Taxes

|  | (1) | (2) | (3) | (4) |
| --- | --- | --- | --- | --- |
| VARIABLES | obesity | diabetes | obesity | diabetes |
|  |  |  |  |  |
| Total grocery sales tax rate | 0.588*** | 0.215** |  |  |
|  | (0.154) | (0.098) |  |  |
| Total restaurant sales tax rate | -0.158 | -0.127 |  |  |
|  | (0.127) | (0.101) |  |  |
| (1+Grocery Tax)/(1+Restaurant Tax) |  |  | 4.760*** | 1.296** |
|  |  |  | (1.169) | (0.571) |
| Grocery stores | -0.244 | -1.113*** | -0.215 | -1.101*** |
|  | (1.262) | (0.406) | (1.258) | (0.408) |
| Fastfood restaurants | -0.377 | 0.289 | -0.377 | 0.292 |
|  | (0.569) | (0.228) | (0.570) | (0.228) |
| Full-service restaurants | 0.080 | -0.063 | 0.091 | -0.062 |
|  | (0.517) | (0.181) | (0.518) | (0.183) |
| Cost per meal | -0.735* | -0.512*** | -0.720* | -0.522*** |
|  | (0.386) | (0.182) | (0.385) | (0.184) |
| White | 11.058 | 1.993 | 11.088 | 1.994 |
|  | (14.889) | (5.913) | (14.926) | (5.901) |
| Black | 53.462** | 23.179*** | 53.523** | 22.997*** |
|  | (24.510) | (7.179) | (24.572) | (7.167) |
| Female | -15.464 | -5.143 | -15.609 | -5.151 |
|  | (17.564) | (7.582) | (17.481) | (7.566) |
| Hispanic | -19.896* | -5.802 | -19.886* | -5.607 |
|  | (10.077) | (5.561) | (10.054) | (5.523) |
| Income per capita | 0.025* | 0.010 | 0.026* | 0.010 |
|  | (0.014) | (0.008) | (0.013) | (0.008) |
| Employees' share of total population | -7.258* | -1.045 | -7.353* | -1.011 |
|  | (3.752) | (1.161) | (3.762) | (1.168) |
| Share of bachelor’s degree or higher of the 25-year- and-over population | -0.032 | -0.016 | -0.032 | -0.016 |
|  | (0.045) | (0.018) | (0.045) | (0.018) |
| Smoking rate | 0.002 | 0.004 | 0.002 | 0.004 |
|  | (0.030) | (0.008) | (0.030) | (0.008) |
| Drinking rate | -0.020 | -0.010 | -0.020 | -0.010 |
|  | (0.023) | (0.013) | (0.023) | (0.014) |
| Drug arrest rate | 4.294*** | -1.055*** | 4.290*** | -1.054*** |
|  | (0.241) | (0.125) | (0.240) | (0.125) |
| DUI | -5.908*** | 1.619*** | -5.903*** | 1.619*** |
|  | (0.384) | (0.176) | (0.384) | (0.178) |
| Constant | 32.161* | 11.305 | 30.258 | 10.356 |
|  | (18.835) | (7.253) | (18.697) | (7.262) |
|  |  |  |  |  |
| Observations | 9,779 | 9,779 | 9,779 | 9,779 |
| R-squared | 0.910 | 0.928 | 0.910 | 0.928 |

Note: Standard errors are in parentheses. *, **, and *** denote statistically significance at the 10%, 5%, and 1% levels, respectively.

**A2. Calculation of Health Burden and Benefit-Cost Ratio** We calculate the aggregate U.S. health burden of grocery tax rates in the year 2016 based on direct costs of treating obesity and diabetes and the cost of mortalities. Direct costs are measured as the medical expenditures for treating people with obesity and for treating people with diabetes; the cost of mortalities is measured as the value of statistical life (VSL). Our preferred estimates of annual expenditures (direct costs only) for treating obesity and diabetes are $1,901 [1] and $9,601 [2], respectively.

The first step to calculate these aggregate health burdens is to calculate the additional cases of people with obesity and diabetes at the county level. These counts are calculated based on multiplying the regression coefficients relating grocery taxes to obesity and diabetes by the grocery tax rate in a county, and then multiplying by the county population. These products deliver county-level estimates of the additional people with obesity and diabetes associated with an increase in the grocery tax rate. Next, we multiply these additional cases of people with obesity and diabetes by our estimates of annual medical expenditures on obesity/diabetes, which deliver estimates of health burdens aggregated at the county level. To recover a national aggregate estimate we aggregate our county-level estimates across all counties with grocery taxes.

Next, we calculate benefit-cost ratios (BCRs) to summarize whether the health benefits associated with reducing the grocery tax by one percentage point are likely to exceed the cost of foregone tax revenues from the reduction. The numerator of the BCR captures the health benefits per person of reducing the grocery tax by one percentage point. This is calculated as the product of 1) the regression coefficient relating grocery taxes to a health outcome, 2) a one percentage point tax reduction, and 3) our preferred estimate for annual expenditures on treatment. The denominator is the cost per person, in terms of foregone tax revenue, of reducing grocery taxes by one percentage point. The average annual food at-home expenditure of U.S. households was $4,363 (USDA ERS, Food Expenditure Series), which translates to $1630.78 per person assuming average household size was 2.6; thus, a one percentage point reduction implies $16.31 per person in foregone annual grocery tax revenue. If the benefit-cost ratio exceeds one, then the marginal benefit exceeds the marginal cost. We complete sensitivity analyses for both the health burden and benefit-cost ratio calculations using different estimates for the direct costs of treating obesity or diabetes for person with the condition.

We found variations among cost estimates; for example, a meta-analysis found that the annual medical expenditures attributable to treating obesity for a person with the condition varies from $1,239 to $2,582 [2]. Therefore, in a sensitivity analysis we consider low and high estimates for these figures, these results are also summarized in Table 4.

The health burden and BCR estimates do not take into account all of the potential adverse impacts of grocery taxes; for example, their effects on indirect costs of obesity or diabetes such as decreases in quality of life or lost work productivity. We also note that obesity and diabetes are related; our estimates of health burden are based on the assumption that they are separate. It is possible that combining the health burden from obesity and diabetes produces an over-estimate. On the other hand, as we have already suggested, the grocery tax might be associated with other adverse effects for which we do not account (as another example, household food insecurity). Not accounting for these other mechanisms would lead to an under-estimate.

REFERENCES

1. American Diabetes Association. Economic Costs of Diabetes in the US in 2017. Diabetes Care 2018;**41**:917

2. Kim DD, Basu A. Estimating the medical care costs of obesity in the United States: systematic review, meta-analysis, and empirical analysis. Value in Health 2016;**19**(5):602-13
